# Autophagy Inhibition Compromises Degradation of Ubiquitin-Proteasome Pathway Substrates

**DOI:** 10.1016/j.molcel.2009.01.021

**Published:** 2009-02-27

**Authors:** Viktor I. Korolchuk, Alicia Mansilla, Fiona M. Menzies, David C. Rubinsztein

**Affiliations:** 1 Department of Medical Genetics, University of Cambridge, Cambridge Institute for Medical Research, Addenbrooke's Hospital, Hills Road, Cambridge CB2 0XY, UK

**Keywords:** PROTEINS

## Abstract

The two main routes that cells use for degrading intracellular proteins are the ubiquitin-proteasome and autophagy-lysosome pathways, which have been thought to have largely distinct clients. Here, we show that autophagy inhibition increases levels of proteasome substrates. This is largely due to p62 (also called A170/SQSTM1) accumulation after autophagy inhibition. Excess p62 inhibits the clearance of ubiquitinated proteins destined for proteasomal degradation by delaying their delivery to the proteasome's proteases. Our data show that autophagy inhibition, which was previously believed to only affect long-lived proteins, will also compromise the ubiquitin-proteasome system. This will lead to increased levels of short-lived regulatory proteins, like p53, as well as the accumulation of aggregation-prone proteins, with predicted deleterious consequences.

## Introduction

The ubiquitin-proteasome system (UPS) and the autophagy-lysosome pathway are the two main routes for eukaryotic intracellular protein clearance. Proteasomes are barrel-shaped, multiprotein complexes that predominantly degrade short-lived nuclear and cytosolic proteins and retrotranslocated, misfolded endoplasmic reticulum proteins. Typically, proteins are targeted for proteasomal degradation after being covalently modified with ubiquitin. This conjugation employs three types of enzymes: E1 (ubiquitin-activating enzyme); E2 (ubiquitin-conjugating enzyme); and E3 (ubiquitin ligase). Often, the ubiquitin itself forms a substrate for further rounds of ubiquitination, resulting in the formation of a polyubiquitin chain. These polyubiquitin chains serve as a recognition signal that allows substrates to be escorted to the proteasome via a set of proteins that, at least in certain cases, includes the chaperone CDC48/p97 (Richly et al., 2005).

Macroautophagy (which we will call autophagy) involves the sequestration of cytoplasm by double-layered membranes to form vesicles called autophagosomes, which ultimately fuse with lysosomes, in which their contents are degraded. The formation of autophagosomes is regulated by two connected ubiquitin-like conjugation systems. The first involves conjugation of Atg12 to Atg5, mediated by the E1-like enzyme Atg7 and the E2-like enzyme Atg10. The second involves conjugation of Atg8 to the lipid phosphatidylethanolamine, which is regulated by Atg7 along with Atg3 as the E2-like enzyme (Xie and Klionsky, 2007).

When autophagy is inactivated in otherwise normal mice by knockout of *atg5* or *atg7*, aggregates and ubiquitinated proteins accumulate in various tissues (Hara et al., 2006; Komatsu et al., 2006). One possibility is that such inclusions form because many different aggregate-prone proteins are autophagy substrates (Rubinsztein, 2006), and these become ubiquitinated either because the proteins are misfolded or because the substrates are comparatively inert and slowly turned over (Komatsu et al., 2006). Because proteasome activity was not affected by *atg7* knockout, it was assumed that UPS function was intact (Komatsu et al., 2006). It was proposed that the accumulation of these ubiquitinated substrates was a direct consequence of autophagy deficiency; in other words, these proteins accumulated because they were normally dependent on autophagy for their clearance (Hara et al., 2006; Komatsu et al., 2005, 2006). Here, we have tested the possibility that autophagy inhibition may impact on flux through the UPS, and may thereby influence clearance of ubiquitinated, short half-life, proteins. Our data show that autophagy inhibition increases the levels of soluble UPS clients by slowing their clearance at a step upstream of proteasome catalytic activity. Decreased clearance of proteasome substrates is associated with the accumulation of p62 after autophagy knockdown, and knockdown of p62 in autophagy-deficient cells normalizes levels of UPS clients. Since increasing p62 levels in autophagy-competent cells to the extent seen in autophagy deficiency also result in impaired clearance of UPS clients, our data strongly argue that the UPS flux deficiency caused by reduced autophagic activity is predominantly mediated by p62.

## Results

### Autophagy Inhibition Impairs Flux through UPS

**Figure 1:**
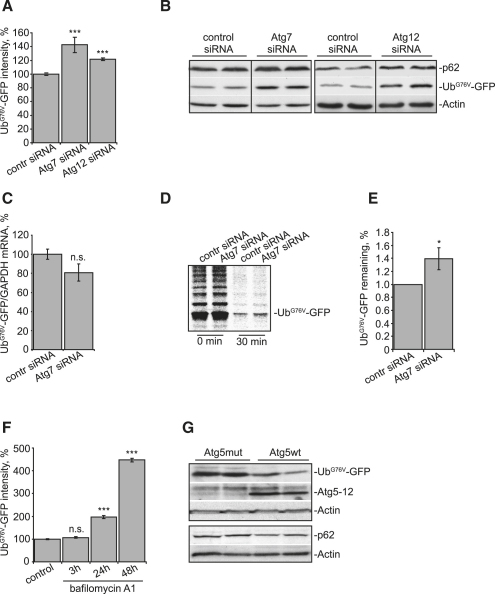
Inhibition of Autophagy Leads to Impairment of Proteasomal Degradation (A and B) siRNA against autophagosomal proteins increases levels of Ub^G76V^-GFP. Ub^G76V^-GFP HeLa cells were transfected with siRNA against two autophagosomal proteins (Atg7 and Atg12), followed by a 72 hr incubation to allow for protein knockdown. GFP fluorescence intensity was quantified by FACS (A), or cells were subjected to immunoblotting (B). (C) Knockdown of Atg7 does not affect mRNA levels of Ub^G76V^-GFP. mRNA from cells treated as in (A) was used to measure amounts of Ub^G76V^-GFP transcript relative to glyceraldehyde 3-phosphate dehydrogenase (GAPDH) by quantitative PCR. (D and E) Knockdown of Atg7 slows degradation of Ub^G76V^-GFP. (D) Levels of [^35^S]methionine Ub^G76V^-GFP were assessed immediately after radioactive pulse (0 min), or following a 30 min chase in the absence of the radiolabel. Bands larger than the main product likely represent different ubiquitinated species. (E) The ratio of [^35^S]Ub^G76V^-GFP at 30 min to 0 min was significantly higher in *atg7* siRNA-treated cells (n = 3). Control values for 30 min/0 min values are normalized to 1, to allow for comparisons of different gels and experiments. In this experiment, the control value at 30 min was 2.27%, whereas that of the Atg7 knockdown was 2.88%. Similar significant findings were obtained in an independent triplicate experiment. (F) A chemical inhibitor of autophagy, bafilomycin A1, increases levels of UPS reporter in a time-dependent manner. Ub^G76V^-GFP HeLa cells were treated with either DMSO (control) for 48 hr or with 100 nM bafilomycin A1 for the indicated periods of time. GFP fluorescence intensity was quantified by FACS. (G) Expression of wild-type (wt), but not mutant, Atg5 in *atg5^−/−^* MEFs reduces levels of UPS reporter. *atg5*^−/−^ MEFs were transfected with Ub^G76V^-GFP and either wild-type or mutant (K130R) Atg5 (1:3 ratio). Cells were lysed 48 hr posttransfection and subjected to immunoblotting. Note, only wild-type, but not mutant, Atg5 forms a functional conjugate with Atg12. For all of the graphs, data are shown as means ± SE for three separate experiments performed in triplicate. ^∗^p < 0.05, ^∗∗∗^p < 0.005, t test; all other comparisons are not significant (n.s.).

We used two experimental paradigms to test if autophagy inhibition may impact on flux through the UPS. In one set of experiments, we inhibited autophagy by siRNA knockdown of two different genes that regulate autophagosome formation (*atg7* and *atg12*) and tested if this affected levels of ubiquitin-primed GFP (Ub^G76V^-GFP). This Ub^G76V^-GFP construct is a ubiquitin-proteasome pathway activity reporter, which normally turns over rapidly and is present at low levels, but accumulates quickly if proteasome activity is impaired (Dantuma et al., 2000). We found that inhibition of autophagy by siRNA knockdown of Atg7 or Atg12 for 72 hr in HeLa cells stably expressing Ub^G76V^-GFP (Ub^G76V^-GFP HeLa) (see Figure S1A available online) led to an increase of GFP fluorescence and Ub^G76V^-GFP protein levels (Figures 1A and 1B), suggesting impairment of proteasomal degradation. Ub^G76V^-GFP accumulation was not due to changes in its mRNA levels (Figures 1C and S1B), and *atg7* siRNA led to impaired clearance of Ub^G76V^-GFP in a pulse-chase experiment (Figures 1D and 1E). Consistent with these data, treatment of cells with the autophagy inhibitors bafilomycin A1 or 3-methyladenenine (3-MA) or the lysosomal enzyme inhibitors pepstatin A/E64d for 24 hr or longer increased Ub^G76V^-GFP levels (Figures 1F and S1C).

In a different experimental design, we used an Atg5-deficient, autophagy-incompetent mouse embryonic fibroblast (MEF) cell line. We transfected these cells with the Ub^G76V^-GFP plasmid together with DNA constructs encoding either wild-type Atg5 protein or the conjugation-deficient (autophagy-incompetent), K130R mutant, Atg5 (Mizushima et al., 2001). We found that the expression of wild-type Atg5 not only rescued autophagic activity of the mutant MEF cell line (manifested by the formation of a Atg5-Atg12 conjugate), but also resulted in reduced levels of Ub^G76V^-GFP, compared to the mutant Atg5 (Figure 1G). Taken together, these data suggest that inhibition of autophagy by siRNAs or chemical inhibitors, or in knockout fibroblasts, results in increased levels of a UPS reporter.

In order to confirm that these observations are not simply due to Ub^G76V^-GFP being an autophagy substrate, we further investigated the degradation routes of this protein. Treatment of cells with proteasome inhibitor for 3 hr led to a clear increase in levels of Ub^G76V^-GFP, whereas treatment with bafilomycin A1 for the same period of time had no effect (Figures S1D–S1F). However, at the same time point, levels of p62, an autophagy substrate, were increased (Figures S1E and S1F). Likewise, trehalose, which induces autophagy (without affecting protein synthesis via mTOR inhibition [Sarkar et al., 2007]), has no effect on the levels of Ub^G76V^-GFP (Figures S1G and S1H).

Longer treatment with bafilomycin A1 and the proteasome inhibitor lactacystin caused no additional increase in Ub^G76V^-GFP protein levels, compared to lactacystin alone (Figure S1I). We believe that Ub^G76V^-GFP is accumulating due to decreased clearance via the UPS, since if autophagy were a significant route for Ub^G76V^-GFP clearance then one would expect an added increase in the levels of Ub^G76V^-GFP when both systems were inhibited. The paradigm we used was to treat cells with bafilomycin A1 for 32 hr with or without lactacystin for the last 8 hr. The absence of additive effects of inhibition of both the proteasome and autophagy would occur in the scenario that autophagy inhibition only significantly altered UPS flux after longer periods, as we see in Figure 1F. These inhibitor data were confirmed, as treatment of cells with the proteasome inhibitor MG132 along with autophagy inhibition with *atg7* siRNA caused no additional increase in Ub^G76V^-GFP protein levels, compared to MG132 alone (Figure S1J).

**Figure 2:**
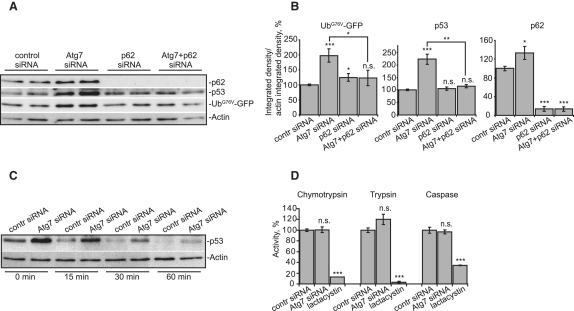
Knockdown of p62 Reduces Elevated Levels of UPS Substrates Caused by Impaired Autophagy (A and B) Ub^G76V^-GFP HeLa cells were transfected with control or *p62* siRNA. After 48 hr, cells were further transfected with control or *atg7* siRNA and incubated for 72 hr. Cells were analyzed by immunoblotting (A), and bands were quantified by densitometry (B). (C) Degradation of endogenous p53 is delayed in cells transfected with siRNA against *atg7*. After Atg7 knockdown for 96 hr, cells were incubated with 50 μg/ml cycloheximide for the indicated periods, then lysed and subjected to immunoblotting. (D) Knockdown of Atg7 does not affect trypsin-, chymotrypsin-, and caspase-like proteolytic activities of the proteasome. Ub^G76V^-GFP HeLa cells were transfected with control or *atg7* siRNA as in (A). A total of 72 hr posttransfection, cells were lysed and proteasome activities measured. Cells treated with the proteasome inhibitor lactacystin were used as a positive control. For all of the graphs, data are shown as means ± SE for three separate experiments performed in triplicate. ^∗^p < 0.05, ^∗∗^p < 0.01, ^∗∗∗^p < 0.005, t test; all other comparisons are not significant (n.s.).

In addition to the effect on the artificial UPS reporter, we found that Atg7 knockdown increased the levels of the endogenous proteasome substrates p53 and β-catenin (Aberle et al., 1997; Haupt et al., 1997; Kubbutat et al., 1997) (Figures 2A, 2B, S2A, and S2B). As with Ub^G76V^-GFP, we confirmed that p53 showed no dependency on autophagy for its degradation. Autophagy induction or short-term autophagy inhibition had no effect on p53 levels (Figures S1E–S1H), whereas this protein rapidly accumulates after proteasome inhibition with MG132 (Figure S1D). Also, there was no additional p53 accumulation in cells treated with *atg7* siRNA and MG132, or bafilomycin A1 and MG132, compared to MG132 alone (Figures S2C and S2D). Whereas β-catenin levels were not increased by 3 hr of bafilomycin A1 treatment, they were decreased by the autophagy enhancer trehalose, and thus this protein may have some dependence on autophagy for its clearance, in addition to the UPS (Figures S1E–S1H).

In order to show that increases in p53 levels do not result from augmented protein synthesis but from slower degradation, we monitored the degradation kinetics of p53 after short-term blocks of translation with cycloheximide, a standard approach used to assay proteasome-mediated degradation (Zhou, 2004). When protein synthesis was blocked, endogenous p53 was turned over more slowly in autophagy-impaired cells, compared to control cells (Figures 2C, S2E, and S2F). (As expected, the turnover of p53 was dramatically impaired by the proteasome inhibitor MG132 [Figure S2G].) In summary, the data presented above reveal that autophagy compromise inhibits the clearance of UPS client proteins, and this may, in part, account for the increases in ubiquitinated proteins in autophagy-deficient mice (Hara et al., 2006; Komatsu et al., 2006).

### p62 Elevation Causes UPS Compromise Due to Autophagy Inhibition

We next focused our attention on potential mechanisms of UPS impairment due to autophagy compromise. Atg7 knockdown did not impair proteasome catalytic activity (Figure 2D), compatible with previously published data in *atg7* knockout mice (Komatsu et al., 2006). This suggested that the UPS deficit due to autophagy compromise lay upstream of the proteasome. As ubiquitinated proteins accumulate under conditions of autophagy knockdown (Hara et al., 2006; Komatsu et al., 2006), we considered that ubiquitination itself was unlikely to be perturbed, but that the problem may have been due to inefficient delivery of ubiquitinated proteins to the proteasome.

Recently, Komatsu et al. (2007) observed that the ubiquitin-binding protein p62 was an autophagy substrate and accumulated when autophagy was blocked, consistent with previous data (Bjorkoy et al., 2005) and our data (Figures 1B, 1G, 2A, 2B, S1E, and S1F). They observed that the formation of aggregates in autophagy-null mice was abrogated when *p62* was knocked out (*p62*-knockout, autophagy-competent mice were normal). Very similar data were recently reported for the *Drosophila p62* ortholog. Although ubiquitinated aggregates in the fly brain were a consequence of mutation of the autophagy gene *atg8a*, this phenotype was attenuated in flies that had double mutations of *atg8a* and the fly ortholog of *p62* (Nezis et al., 2008). The interpretation of these studies in mice and flies was that p62 levels increased when autophagy was knocked out, and that the increase directly enhanced the aggregation process itself, although the mechanisms remained unclear (Komatsu et al., 2007; Nezis et al., 2008).

In light of our data presented above, we considered an additional possibility: autophagy blockade causes accumulation of p62, which compromises the clearance of ubiquitin-proteasome pathway substrates. Thus, we tested if siRNA knockdown of p62 could rescue the effects of autophagy inhibition on Ub^G76V^-GFP accumulation. Indeed, *p62* siRNA reduced Ub^G76V^-GFP and p53 levels in cells in which autophagy was compromised (Figures 2A and 2B). Importantly, p62 knockdown almost completely normalized the effects of autophagy compromise of the UPS client levels (Figures 2A and 2B), suggesting that it was the major mediator of the effect.

The dependence on p62 for the accumulation of these substrates after autophagy inhibition is not simply due to the fact that the proteins are sequestered into the aggregates of p62 that form when its degradation is impaired, since all the blots we have used have assayed the levels of soluble substrates (see Experimental Procedures). This was confirmed when we separated cell lysates into soluble and insoluble fractions and observed that p62 increases in both soluble and insoluble fractions when its degradation is impaired with either UPS or autophagy inhibitors, whereas Ub^G76V^-GFP and p53 predominantly accumulate in the soluble fraction (Figure S3A). Also, neither of these substrates colocalize with the p62 aggregates formed in cells treated with bafilomycin A1 (Figure S3B).

These data suggest that the extent to which UPS substrates accumulate after autophagy inhibition is directly due to the levels of p62. Thus, one will get greater perturbation of the UPS the longer one inhibits autophagy (until the system becomes saturated). This is consistent with what is seen when we use a chemical inhibitor of autophagy (Figure 1F). Indeed, the elevations of p62 in the autophagy-deficient mice observed by Komatsu et al. (2007) are huge (at least 10-fold increase), as is the increase in ubiquitinated proteins in vivo.

### p62 Overexpression at Levels Similar to Those in Autophagy-Compromised Cells Inhibits UPS

**Figure 3:**
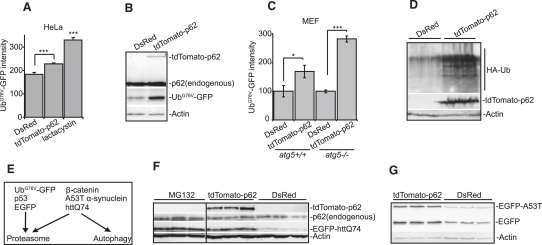
Overexpression of p62 Inhibits Degradation of Proteasomal Substrates (A) p62 overexpression increases levels of Ub^G76V^-GFP. HeLa cells were cotransfected with Ub^G76V^-GFP and either DsRed or tdTomato-p62 (1:3 ratio) and incubated for 48 hr. A separate set of Ub^G76V^-GFP/DsRed-transfected cells were treated with 10 μM lactacystin 16 hr prior to analysis as a positive control. The GFP fluorescence intensity of double-positive green/red cells was quantified by FACS. (B) Levels of Ub^G76V^-GFP are increased in the presence of overexpressed p62. SK-N-SH cells were transfected as in (A). After 48 hr, cells were harvested and immunoblotted. The levels of exogenous p62 appear to be low because they are measured in the whole cell population from a transient transfection experiment, where only a proportion of cells express the transgene. (C) p62 overexpression causes accumulation of Ub^G76V^-GFP in wild-type and autophagy-deficient cells. *atg5^+/+^* and *atg5^−/−^* MEFs were transfected as in (A), and the GFP fluorescence intensity of double-positive green/red cells was analyzed by FACS. We normalized the levels in both *atg5^+/+^* and *atg5^−/−^* MEFs in control conditions to 100% to facilitate comparisons; however, the levels of Ub^G76V^-GFP are higher in the autophagy-deficient MEFs. (D) Overexpression of p62 leads to accumulation of ubiquitinated proteins. SK-N-SH cells were transfected with HA-Ub and either DsRed or tdTomato-p62 (1:1 ratio), incubated for 48 hr, and analyzed for levels of HA-Ub-labeled proteins by immunoblotting. (E) Schematic diagram of degradation pathways for proteins used in this study. (F) p62 increases levels of soluble polyQ. HeLa cells were transfected with EGFP-httQ74 and either DsRed or tdTomato-p62 (1:3 ratio) and were incubated for 48 hr. A separate set of EGFP-httQ74/DsRed-transfected cells was treated with the proteasomal inhibitor MG132, as a positive control. (G) p62 increases levels of soluble mutant α-synuclein (EGFP-A53T) and EGFP. HeLa cells were transfected with EGFP-A53T, EGFP, and either DsRed or tdTomato-p62 (1:1:3 ratio) and were incubated for 48 hr prior to immunoblotting. For all of the graphs, data are shown as means ± SE for three separate experiments performed in triplicate. ^∗^p < 0.05, ^∗∗∗^p < 0.005, t test; all other comparisons are not significant (n.s.).

These data suggested that p62 overexpression in normal cells should mimic the effects of autophagy blockade on Ub^G76V^-GFP levels. Indeed, Ub^G76V^-GFP accumulated in cells overexpressing p62 (Figures 3A and 3B) at levels that are of a similar magnitude to what we see with autophagy inhibition (Figure 1A). Ub^G76V^-GFP also accumulated in autophagy-deficient cells overexpressing p62 (Figure 3C), suggesting that the p62 overexpression effects were autophagy independent. In agreement with this, overexpression or knockdown of p62 did not affect autophagosome numbers (as monitored by levels of the specific autophagosome marker, lipid-conjugated microtubule-associated protein light chain 3/LC3-II), and p62 knockdown did not affect autophagic flux (LC3-II levels measured in cells in which autophagosome degradation was inhibited [Sarkar et al., 2007]) (Figure S4). Likewise, Komatsu et al. (2007) described that *p62* knockout mice have no abnormalities in autophagy.

Overexpression of p62 increased levels of ubiquitinated proteins (Figure 3D), a phenomenon also seen with autophagy knockdown (Hara et al., 2006; Komatsu et al., 2006). We also tested the effect of p62 overexpression on other polypeptides, such as mutant polyglutamine(polyQ)-expanded huntingtin fragment (EGFP-httQ74), mutant α-synuclein associated with familial Parkinson's disease (EGFP-A53T), and EGFP alone, all of which are substrates for the UPS (Rubinsztein, 2006) (Figure 3E) and accumulate in cells treated with proteasome inhibitors (Ravikumar et al., 2002; Webb et al., 2003) (Figure 3F). Overexpression of p62 resulted in increased protein levels of all of these proteins (Figures 3F and 3G).

**Figure 4:**
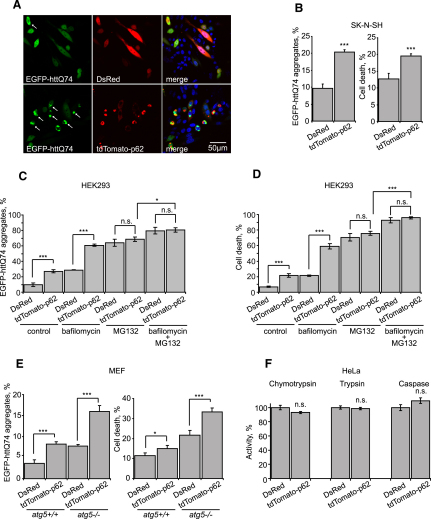
Overexpression of p62 Increases Aggregation and Toxicity of httQ74 (A and B) p62 overexpression increases polyQ aggregation and toxicity. SK-N-SH cells were cotransfected with EGFP-httQ74 and DsRed (control) or tdTomato-p62 at 1:3 ratios. Representative images of SK-N-SH cells are shown in (A). White arrows indicate cells containing EGFP-httQ74 aggregates. (B) The percentage of green/red double-positive cells with EGFP-httQ74 aggregates or apoptotic nuclear morphology was assessed 24 hr posttransfection. (C and D) p62 increases polyQ aggregation and toxicity in cells in which autophagy, but not the proteasome, is inhibited. HEK293 cells were transfected as in (A), followed by a 24 hr bafilomycin A1 and/or MG132 treatment. The percentage of transfected cells (red/green positive) with polyQ aggregates and cell death were quantified. (E) p62 increases toxicity and aggregation of polyQ in autophagy-deficient *atg5*^−/−^ cells. *atg5^+/+^* and *atg5^−/−^* MEFs were transfected with EGFP-httQ74 and either DsRed or tdTomato-p62 (1:3 ratio). The percentage of green/red double-positive cells with EGFP-httQ74 aggregates or apoptotic nuclear morphology was assessed at 48 hr post-transfection. (F) Overexpression of p62 does not affect trypsin-, chymotrypsin-, and caspase-like proteolytic activities of the proteasome. HeLa cells were transfected with either DsRed or tdTomato-p62 and incubated for 48 hr. Fluorescent cells were sorted and lysed, and proteasome activities were measured. For all of the graphs, data are shown as means ± SE for three separate experiments performed in triplicate. ^∗^p < 0.05, ^∗∗∗^p < 0.005, t test; all other comparisons are not significant (n.s.).

As the formation of aggregates/inclusions is a concentration-dependent phenomenon (Tartaglia et al., 2007) and correlates linearly with expression levels of EGFP-httQ74 (Narain et al., 1999), all other factors being equal, we carefully assessed if p62 overexpression also affected the levels of aggregate-prone, disease-associated proteins and if this effect was autophagy independent. Note that we have used the huntingtin aggregation as an additional surrogate for substrate accumulation in this context. In our hands, httQ74 does not affect activity of the UPS, as assessed by the levels of Ub^G76V^-GFP (Figure S5A). p62 overexpression led to an increase in the proportions of cells expressing EGFP-httQ74 with aggregates, and this correlated with an increase in cell death in these cells (Figures 4A–4D and S5B). p62 overexpression led to a similar increase in aggregation in cells in which autophagy was compromised due to bafilomycin A1, 3-MA, or *atg5* knockout (Figures 4C, 4E, and S5C). As the EGFP-httQ74 aggregation and clearance is dependent on both the UPS and autophagy (Ravikumar et al., 2002) (Figure 4C) and the effects of p62 on EGFP-httQ74 aggregation are preserved in autophagy-deficient cells, we considered that p62 overexpression may be compromising clearance via the UPS route. Consistent with this hypothesis, no further increase in aggregation is seen in MG132- or lactacystin-treated cells overexpressing p62 (Figures 4C and S5C). p62 overexpression also did not increase httQ74 aggregate numbers when both autophagy and proteasome activities were inhibited (Figures 4C and S5D). Moreover, aggregation of an ubiquitin-tagged, proteasome-targeted polyQ was increased by overexpression of p62 in a proteasome-dependent manner (Figure S5E). Thus, p62 overexpression leads to the accumulation of a specific ubiquitin-proteasome pathway reporter (Ub^G76V^-GFP), a proteasome-targeted substrate (Ubi-R-Q112-EGFP), and aggregate-prone (mutant huntingtin, EGFP-httQ74) and non-aggregate-prone (EGFP) UPS clients in wild-type cells, or in cells in which autophagy is compromised. However, p62 overexpression does not have these effects in cells treated with a proteasome inhibitor. We conclude that increased levels of p62 result in perturbation of the ubiquitin-proteasome pathway. Interestingly, elevated p62 levels have been reported in model systems of neurodegenerative diseases including Huntington's disease (Gal et al., 2007; Nagaoka et al., 2004), and this may be sufficiently high to contribute to the accumulation of ubiquitinated substrates that has been recently reported in this condition (Bennett et al., 2007).

**Figure 5:**
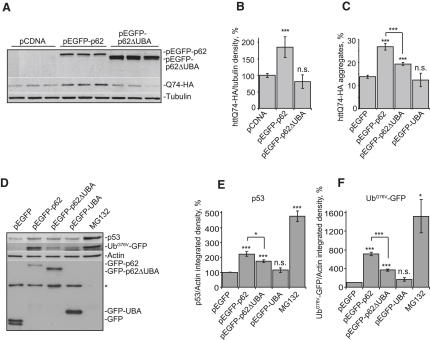
p62 Increases Levels of Proteasome Substrates in a UBA-Dependent Manner (A and B) Overexpression of full-length, but not truncated, p62 increases levels of soluble polyQ. HeLa cells were transfected with httQ74-HA and either pcDNA (control), pEGFP-p62, or pEGFP-p62ΔUBA at 1:3 ratios and were incubated for 48 hr. (B) Cells were immunoblotted, and bands were quantified by densitometry. (C) Effect of p62 overexpression on polyQ aggregation is dependent on UBA. SK-N-SH cells were cotransfected with httQ74-HA and either pEGFP-C1 (control), pEGFP-p62, pEGFP-p62ΔUBA, or pEGFP-UBA at 1:3 molar ratios. A total of 24 hr posttransfection, cells were immunostained for HA. The percentage of double-positive cells with httQ74 aggregates was counted. (D–F) Overexpression of p62 increases levels of p53 and Ub^G76V^-GFP. HeLa cells were transfected with Ub^G76V^-GFP and either pEGFP-C1 (control), pEGFP-p62, pEGFP-p62ΔUBA, or pEGFP-UBA at 1:3 molar ratios. A total of 24 hr posttransfection, cells were analyzed by immunoblotting. (D) Cells transfected only with Ub^G76V^-GFP were incubated with MG132 for 3 hr prior to lysis, as a positive control (D). The asterisk denotes a nonspecific band. (E and F) Levels of endogenous p53 and Ub^G76V^-GFP were quantified by densitometry. For all of the graphs, data are shown as means ± SE for three separate experiments performed in triplicate. ^∗^p < 0.05, ^∗∗∗^p < 0.005, t test; all other comparisons are not significant (n.s.).

Overexpression of p62 did not lead to a compromise of proteasome catalytic activity (Figure 4F). Instead, p62 must be acting upstream of the proteasome, as its knockdown did not rescue increased levels of Ub^G76V^-GFP caused by direct inhibition of proteasomal activity (Figures S6A and S6B). As p62 binds avidly to ubiquitinated proteins through its ubiquitin-associated (UBA) domain (Seibenhener et al., 2007), we speculated that p62, when present at elevated levels, acts upstream of the proteasome by binding ubiquitinated proteins, possibly by preventing them from shuttling to the proteasome. Consistent with this, deletion of the UBA from p62 impaired its ability to cause httQ74 accumulation/aggregation (Figures 5A–5C), and it had a similar effect on Ub^G76V^-GFP and p53 accumulation (Figures 5D–5F). Since the deletion of the UBA domain did not completely abolish the effects of p62 overexpression, it is also possible that elevated levels of p62 may impair clearance of proteasome substrates both by UBA-dependent and UBA-independent mechanisms. Overexpression of the UBA alone had no effect on httQ74 aggregation or on p53 or Ub^G76V^-GFP levels (Figures 5C–5F). This may be because ubiquitin binding of the UBA domain is dependent on its protein context within p62 (Seibenhener et al., 2007), or because p62 dimerization or its ability to interact with the proteasome are required for the effects we have observed (Seibenhener et al., 2007).

### p97 Rescues Effects of p62 Overexpression

**Figure 6:**
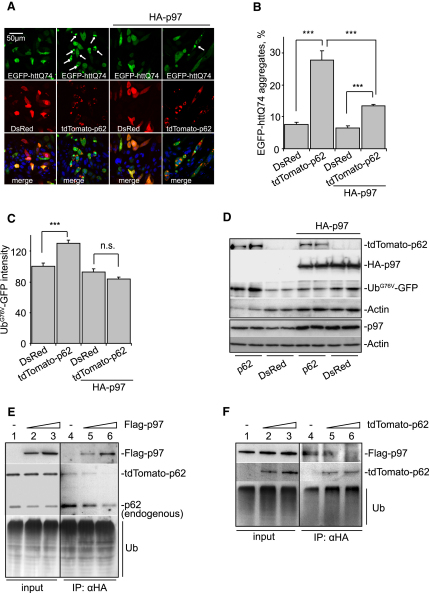
p97 Alleviates the Effects of p62 Overexpression (A and B) p97 overexpression rescues increased polyQ aggregation caused by p62 overexpression. SK-N-SH cells were cotransfected with EGFP-httQ74, along with either DsRed or tdTomato-p62, and either pcDNA3.1 or HA-p97 at 0.5:1:1 ratio. (A) After 24 hr of incubation, cells were imaged. White arrows indicate cells containing EGFP-httQ74 aggregates. (B) The percentage of green/red double-positive cells with EGFP-httQ74 aggregates was quantified. (C and D) p97 overexpression reduces elevated Ub^G76V^-GFP levels caused by p62 overexpression. SK-N-SH cells were transfected as in (A), but EGFP-httQ74 was replaced with Ub^G76V^-GFP. A total of 48 hr posttransfection, Ub^G76V^-GFP levels were assessed either by FACS (C) or immunoblotting (D). (E) p97 competes with p62 for ubiquitin binding. HeLa cells were cotransfected with HA-Ub, tdTomato-p62, and either pcDNA3.1 (lanes 1 and 4) or Flag-p97 at a p62:p97 molar ratio of 1:1 (lanes 2 and 5) or 1:2 (lanes 3 and 6). A total of 24 hr posttransfection, MG132 was added and cells were incubated for an additional 24 hr. Cells were subjected to immunoprecipitation with anti-HA antibody. Lysates (input) and immunoprecipitated samples (IP: αHA) were probed with antibodies against Flag epitope, p62, or ubiquitin. (F) p62 prevents binding of p97 to ubiquitinated proteins. HeLa cells were cotransfected with HA-Ub, Flag-p97, and either pcDNA3.1 (lanes 1 and 4) or tdTomato-p62 at a p97:p62 DNA molar ratio of 1:1 (lanes 2 and 5) or 1:2 (lanes 3 and 6). A total of 24 hr posttransfection, MG132 was added for an additional 24 hr. Cells were subjected to immunoprecipitation as in (E). For all of the graphs, data are shown as means ± SE for three separate experiments performed in triplicate. ^∗∗∗^p < 0.005, t test; all other comparisons are not significant (n.s.).

One way that at least certain ubiquitinated substrates are shuttled to the proteasome is by another ubiquitin-binding protein, p97 (Richly et al., 2005). *p97* mutations in humans result in a syndrome associated with inclusion body formation and impaired ER-associated degradation of ubiquitinated proteins (Weihl et al., 2006). Additionally, knockdown of p97 compromises the clearance of Ub^G76V^-GFP and polyglutamine aggregates in normal cells (Kobayashi et al., 2007; Wojcik et al., 2006). Interestingly, p97 overexpression abrogated the effects of p62 overexpression on polyQ aggregation and on Ub^G76V^-GFP accumulation (Figures 6A–6D). p97 overexpression prevented p62 from binding ubiquitinated proteins (Figure 6E). Similarly, p62 efficiently displaced p97 from complexes with ubiquitinated proteins (Figure 6F). This suggests that p62 overexpression may act, at least in part, by preventing ubiquitinated proteins from binding to the machineries that shuttle them to or into the proteasome.

### Role of p62 in Aggregate Formation

**Figure 7:**
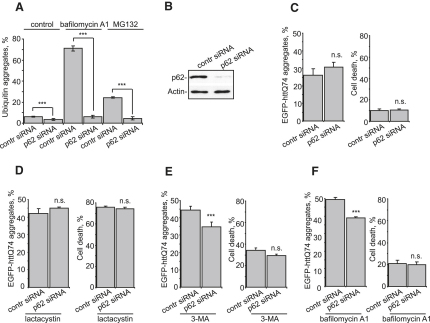
Effect of p62 on Formation of Ubiquitinated Aggregates (A) p62 knockdown reduces ubiquitin-positive aggregates. HeLa cells were transfected with control or *p62*-specific siRNA, followed by a 72 hr incubation. Cells were treated with bafilomycin A1 or MG132 for the final 16 hr, before immunostaining with anti-ubiquitin antibody. The percentage of cells containing ubiquitin-positive aggregates was analyzed. (B) Efficiency of siRNA knockdown. HEK293 cells were transfected as in (A). Cells were analyzed by immunoblotting for endogenous p62. (C) Knockdown of p62 does not affect polyQ aggregation and toxicity. HEK293 cells were transfected with siRNA as in (A). Cells were then retransfected with the same amount of siRNA together with EGFP-httQ74 and incubated for an additional 24 hr before polyQ aggregation and cell death were assessed. (D) Knockdown of p62 does not affect polyQ aggregation and toxicity in the presence of a proteasomal inhibitor. Cells were retransfected as in (C) and incubated for 24 hr in the presence of lactacystin before polyQ aggregation and cell death were assessed. (E and F) Knockdown of p62 decreases the number of polyQ aggregates in cells with inhibited autophagy. Cells were transfected as in (C), followed by a 24 hr incubation with 3-MA (E) or bafilomycin A1 (F). polyQ aggregation and cell death were assessed. For all of the graphs, data are shown as means ± SE for three separate experiments performed in triplicate. ^∗∗∗^p < 0.005, t test; all other comparisons are not significant (n.s.).

Previous studies have suggested that p62 is largely required for the formation of aggregates of endogenous, “wild-type” proteins under conditions of autophagy (or proteasome) impairment (Komatsu et al., 2007; Nezis et al., 2008; Wooten et al., 2006). We confirmed these findings by showing that the numbers of ubiquitinated aggregates, which form in normal cells after autophagy or proteasome inhibition, are dramatically reduced when p62 is knocked down (Figure 7A). Since all of the ubiquitinated aggregates also contain p62 (Figure S7) (Komatsu et al., 2007; Nezis et al., 2008), this phenomenon may be explained if p62 is the main component of these aggregates and also happens to be a protein that binds ubiquitin.

However, we do not think that p62 is required for the formation of all ubiquitinated aggregates. This stems from our observations of mutant huntingtin aggregation. It appears that the httQ74 aggregates are distinct from the ubiquitinated, p62-positive structures that form when autophagy is compromised (Figures S8A and S8B), even though most of the large httQ74 inclusions and all of the p62 inclusions were decorated by anti-ubiquitin antibodies (Figures S7 and S8). In our hands, p62 knockdown did not affect mutant huntingtin aggregation in normal conditions or when proteasome was inhibited (Figures 7B–7D). The latter would be expected if p62 seeded aggregate formation. However, we observed a reduction of httQ74 aggregation by p62 knockdown in autophagy-impaired conditions (Figures 7E and 7F). Under these conditions, p62 accumulates and would therefore inhibit proteasomal degradation, which is relieved by p62 knockdown. These data are in agreement with our observations that p62 overexpression increases polyglutamine aggregation in normal conditions, while having no effect in MG132- and lactacystin-treated cells (as shown above in Figures 4C, S5B, and S5C), where its accumulation can inhibit the UPS to no greater an extent. These observations taken together suggest that p62 only affects httQ74 aggregation via inhibition of UPS flux rather than through seeding of aggregates.

## Discussion

Our data show that after autophagy is inhibited, p62 accumulates, causing impaired delivery of UPS substrates to the proteasome. The proteins that accumulate after autophagy inhibition are well-validated UPS substrates that show dramatic accumulation after short incubations with proteasome inhibitors; however, no such effect is seen with autophagy inhibition until around 24 hr. This suggests that these substrates are quickly cleared by the proteasome, and that the contribution of autophagy to their clearance in normal cells is negligible (although we cannot say this is nonexistent, as any cytosolic protein will be theoretically accessible to autophagosomes).

We can exclude the model that the effects we observed on these UPS substrates after long-term autophagy inhibition is because they are also cleared by autophagy, since the effects are p62 dependent. p62 knockdown protects against the accumulation of these UPS substrates in autophagy-deficient cells (so autophagy cannot be playing a role in the p62-dependent effects), and p62 overexpression leads to the accumulation of all of these substrates in both wild-type and autophagy-deficient cells (again arguing that the effects of p62 must be autophagy independent in this context). Thus, the p62-dependent accumulation of the UPS substrates in autophagy-deficient cells is due to a compromise in the UPS system.

The excess p62 compromises the UPS not by inhibiting proteasome activity, but instead by delaying the delivery of ubiquitinated substrates to the proteasome. After “long” periods of autophagy inhibition, p62 accumulates, leading to a build-up of UPS substrates. This has important consequences for understanding the biological effects of autophagy inhibition, since some of the effects are due to secondary compromise of UPS flux and may be analogous to what one would see with mild proteasome inhibition. The inhibition of UPS flux by autophagy compromise is unexpected, as autophagy upregulation has been reported as a compensatory response after proteasome inhibition (Iwata et al., 2005; Pandey et al., 2007).

The phenomenon of p62 accumulation leading to impaired UPS flux has parallels with the observation that the UBA domain from Rad23 sequesters polyubiquitin chains on proteins and inhibits their degradation by purified proteasomes (Raasi and Pickart, 2003). Similarly, in yeast, overexpression of Dsk2p leads to the accumulation of polyubiquitin chains in an UBA-domain-dependent manner (Funakoshi et al., 2002). Overexpression of the mammalian homologs of Dsk2 (hPLIC1 and hPLIC2) leads to impaired clearance of proteasome substrates such as p53 (Kleijnen et al., 2000), which the authors attribute to perturbation of a step between ubiquitination and degradation. Since p97 overexpression abrogated the effects of p62 overexpression on polyQ aggregation and on Ub^G76V^-GFP accumulation (Figures 6A–6D) and overexpression prevented p62 from binding ubiquitinated proteins (Figure 6E), we favor a model in which p62 overexpression may act, at least in part, by preventing ubiquitinated proteins from binding to the machineries that shuttle them to or into the proteasome. However, we cannot exclude additional roles for p97, as it is a protein with multiple functions (Boyault et al., 2007; Ye, 2006). Likewise, although our data suggest that p62 is not required for autophagosome formation or for httQ74 clearance, and Komatsu et al. (2007) showed that loss of p62 did not compromise lysosome-dependent clearance of proteins under normal or serum-starved conditions (largely autophagy dependent), we cannot exclude that p62 may be involved in autophagic clearance of certain substrates. Additionally, we cannot rule out that autophagy may be crucial for the clearance of certain cytosolic proteins that may become ubiquitinated and aggregate prone when autophagy is compromised. However, these possible roles for p62 are distinct from the thrust of our study.

As many UPS substrates, like p53 and β-catenin, mediate toxicity when their clearance is compromised, our data may help to explain some of the toxicity seen when autophagy is impaired. Autophagy inhibition in cell models results in a two-fold increase in levels of (soluble) p53 and β-catenin (Figures 2A–2C, S2A, and S2B). This is within the same order of magnitude as the increase of p53 and β-catenin resulting from direct inhibition of proteasomal activity (Aberle et al., 1997; Izumi et al., 2001). These changes are physiologically significant and will result in clear perturbations to the cells. The extent of UPS compromise may be more severe in autophagy-compromised mice or in cells in which autophagy is blocked for longer periods (Figure 1F). In the *atg7* knockout mice (Komatsu et al., 2007), p62 levels are elevated to much higher levels than we observed with short-term (and partial) knockdown of autophagy genes.

Our data suggest that autophagy inhibition will have two distinct effects on cells. Here, we describe that this leads to p62 accumulation, which, in turn, impairs flux through the UPS. This mechanism can also account for the accumulation of many of the soluble ubiquitinated proteins in animal models with loss of autophagy (Komatsu et al., 2007). The second phenomenon is that some of the p62 that accumulates after autophagy (or proteasome) inhibition is in ubiquitinated aggregates (as p62 is a ubiquitin-binding protein). This process does not explain the accumulation of UPS substrates seen in the soluble fraction. Likewise, httQ74 aggregates were distinct from the p62 inclusions in autophagy-compromised cells. Indeed, our data suggest that the aggregation of a disease-associated protein like httQ74 is not altered if p62 is knocked down, which contrasts with the ubiquitinated aggregates formed in cells in which the proteasome or autophagy are inhibited, suggesting mechanistic differences between these two types of inclusions. Finally, our data suggest that great care needs to be exercised when attempting to infer that a phenomenon that occurs in autophagy-deficient cells is primarily due to impaired degradation of autophagy substrates, as such cells will also have compromised clearance of UPS clients.

## Experimental Procedures

See the Supplemental Data for additional information on reagents, cell culture, transfection, immunoprecipitation, western blot, and quantitative PCR, all of which were carried out by following standard methods.

### Aggregation and Cell Death Assays

Cells were fixed for 20 min in 4% formaldehyde at room temperature. EGFP httQ74 aggregates were detected by direct fluorescence. httQ74-HA was detected by indirect immunofluorescence by using anti-HA antibody (Covance Laboratories, 1:500) and anti-mouse Alexa 594 secondary antibody (Invitrogen, 1:500). Ubiquitin aggregates were detected by using anti-ubiquitin antibody (Dako, 1:1000) and anti-rabbit Alexa 594 secondary antibody (Invitrogen, 1:500). Slides were mounted in Citifluor (Citifluor, Ltd.) containing 4′,6-diamidino-2-phenylindole (DAPI; 3 μg/ml). Transfected cells were scored by using an Eclipse E600 fluorescence microscope (plan-apo 60×/1.4 oil immersion lens) (Nikon). Cell death was measured as the number of nuclei demonstrating apoptotic morphology (fragmentation or pyknosis). We scored at least 200 transfected cells per slide in triplicate; the scorer was blinded to treatment. Images were acquired on a Zeiss LSM510 META confocal microscope (63× 1.4NA plan-apochromat oil immersion lens) by using Zeiss LSM510 v3.2 software (Carl Zeiss), and Adobe Photoshop 6.0 (Adobe Systems) was used for subsequent image processing.

### Cycloheximide and [^35^S]Methionine Pulse-Chase Experiments

The turnover of p53 in the cycloheximide chase experiments was assessed in HeLa cells transfected with 50 nM control or specific siRNA against Atg7 twice during the period of 96 hr, to allow for protein knockdown. Protein synthesis was then blocked by addition of complete medium containing 50 μg/ml cycloheximide. Cells were lysed at the indicated time points, and p53 levels in the soluble fractions were assessed by western blot.

For [^35^S]methionine pulse-chase experiments, Ub^G76V^-GFP HeLa cells were transfected with siRNA as for the cycloheximide experiments. Cells were incubated for 2 hr in media lacking cysteine and methionine, followed by 30 min labeling with 1.85 MBq (50 μCi) per ml of [^35^S]methionine. Cells were lysed either immediately or following a 30 min incubation in complete medium containing no [^35^S]methionine (chase). Ub^G76V^-GFP was immunoprecipitated by using anti-GFP antibody (Clontech, 1:200) as described in the Supplemental Data and was separated on SDS-PAGE, and gels were dried and exposed to the phosphoimager screen overnight. Incorporation of [^35^S] was measured by using a Storm 860 phosphorimager (GE Healthcare).

### Statistical Analysis on Immunoblots

Densitometry analysis of immunoblots was done by Scion Image Beta 4.02 software (Scion Corporation) from three independent experiments (n = 3). The control condition was set to 100%, and the error bars denote SEM.

### Proteasome Assay

Cells transfected with pDsRed-C1 and pDest-tdTomato-p62 were sorted by a MoFlo Cell Sorter (Dako) 48 hr after transfection, then lysed in UPS lysis buffer (10 mM Tris [pH 7.5], 1 mM EDTA, 20% glycerol, and 0.5% NP40). Cells transfected with control or Atg7 siRNA for 72 hr, or cells treated with 10 μM lactacystin for 16 hr, were lysed in UPS lysis buffer. Proteasome activity was determined by using proteasome assay buffer (Biomol) with Boc-LSTR-7-AMC (Sigma), Suc-LLVY-AMC (Biomol), and Z-LLE-AMC (Biomol) as substrates for the trypsin, chymotrypsin, and caspase-like activities of the proteasome, respectively. Data reflect the kinetics of the linear phases of the curves of fluorogenic substrate production measured with a Cytofluor multiwell plate reader (PerSeptive Biosystems).

## Supplemental Data

Document S1.Supplemental Experimental Procedures and Eight Figures
