# Supplementary material for: Autophagy Inhibition Compromises Degradation of Ubiquitin-Proteasome Pathway Substrates
Source: Mol Cell. 2009 Feb 27;33(4):517–27. doi: 10.1016/j.molcel.2009.01.021 (PMC2669153; doi:10.1016/j.molcel.2009.01.021)

Molecular Cell, Volume *33*

Supplemental Data

Autophagy Inhibition Compromises Degradation of Ubiquitin-Proteasome Pathway Substrates

Viktor I. Korolchuk, Alicia Mansilla, Fiona M. Menzies, and David C. Rubinsztein

Supplemental Experimental Procedures

Reagents

HD gene exon 1 fragment with 74 polyQ repeats in pEGFP-C1 (Clontech) (EGFP-httQ74) or in pHM6 (Roche Diagnostics) (httQ74-HA), and pEGFP-α-synucleinA53T (EGFP-A53T) were described previously (Narain et al., 1999; Sarkar et al., 2008). pDest-tdTomato-p62 (tdTomato-p62), pEGFP-p62, pEGFP-p62ΔUBA and pEGFP-UBA (Bjorkoy et al., 2005), pEGFP-Ub^{G76V} (Ub^{G76V}-EGFP) (Dantuma et al., 2000), Ubi-R-Q112-EGFP (Verhoef et al., 2002), pcDNA.3-HA-p97 (HA-p97), pcDNA.3-FLAG-p97 (FLAG-p97) (Boyault et al., 2006), pcDNA3.1-HA-Ubiquitin (HA-Ub) (Li et al., 2004) and wild-type and K130R Atg5 (Mizushima et al., 2001) plasmids were kindly provided by T. Johansen, N. Dantuma, V. Deretic, S. Khochbin, C. Patterson and N. Mizushima, respectively. pDsRed-C1 (DsRed) was from Clontech, pcDNA3.1 was from Invitrogen, all other reagents were from Sigma.

Cell culture, drug treatment, immunocytochemistry and FACS analysis

Human neuroblastoma cells (SK-N-SH), Human Embryonic Kidney (HEK293), human cervical carcinoma cells (HeLa), and wild-type Atg5 ($Atg5^{+/+}$) and Atg5-deficient ($Atg5^{-/-}$) mouse embryonic fibroblasts (MEFs, kind gift from N. Mizushima) were grown in DMEM medium supplemented with 10% fetal calf serum, penicillin/streptomycin and 2 mM L-glutamine at 37°C in 5% CO_2. Ub^{G76V}-GFP-expressing stable HeLa cell line (kind gift from N.P. Dantuma) was maintained in medium containing 0.5 mg/ml G418. Transfections were performed using Lipofectamine or Lipofectamine 2000 (Invitrogen) according to the manufacturer's instructions. siRNA transfections were carried out using 50 nM control (non-targeting SMARTpool), or specific ON-TARGETplus SMARTpool (Dharmacon). Cells were treated with 10 µM lactacystin (Biomol), 10 µM MG132, 400 nM bafilomycin A1 (Millipore) or 10 mM 3-MA for 24 h (unless otherwise indicated) in growth medium prior to analysis. Cycloheximide was used at 50 µg/ml for the indicated periods of time. Mean fluorescence intensity was quantified by FACS 48 or 72 h after transfection using CyAn ADP (Dako).

Immunoprecipitation and Western blotting

Cells were lysed in IP buffer (20 mM tris pH7.4, 150 mM NaCl, 2 mM MgCl, 0.5% NP-40 with protease inhibitor cocktail (Roche). 100-500 µg of each lysate was incubated for 2 h at 4°C with antibody and for a further 2 h with Protein G-coupled magnetic beads (Invitrogen). Following

incubation, beads were washed 3 times with IP buffer, eluted for 5 minutes in 0.2 M glycine pH 2.5. Alternatively, beads were boiled in SDS-PAGE loading buffer for 5 min and subjected to SDS-PAGE electrophoresis.

For Western blotting analysis, cells were lysed in RIPA buffer (150 mM NaCl, 1% NP-40, 0.5% NaDoC, 0.1% SDS, 50 mM tris-HCl, pH 7.4, supplemented with complete protease inhibitor cocktail (Roche)) for 20 min on ice, followed by centrifugation for 10 min at 13,000 xg to remove insoluble proteins. Concentration of protein in soluble fraction was measured by Bradford assay (BioRad) and 10-40 μg of protein was run on SDS-PAGE gels and transferred to PVDF membrane. The blots were incubated with the following antibodies: anti-p62 (BD Transduction Laboratories, 1:2000 or Progen Biotechnik, 1:1000), anti-GFP (Clontech, 1:2000), anti-actin (1:2000), anti-Flag (1:1000), anti-HA (Covance, 1:1000), anti-LC3 (Novus Biologicals, 1:2000), anti-Atg5 (Novus Biologicals, 1:1000), anti-p53 (Cell Signaling, 1:1000), anti β-catenin (Zymed Laboratories, 1:500), or anti-Ubiquitin (Chemicon, 1:1000) in 5% non fat milk in PBS (10 mM phosphate buffer containing 140 mM NaCl and 3 mM KCl, pH 7.4) at 4°C overnight, washed twice with PBS and incubated with the secondary HRP-conjugated antibodies (Roche, anti-mouse and anti-rabbit 1:5000 or Dako, anti-guinea pig 1:1000) for 1 h at room temperature. The blots were washed extensively in PBS and the signal was detected by autoradiography using the ECL Western blotting kit (GE Healthcare).

Quantitative PCR

RNA from Ub^{G76V}GFP Hela transfected with control or Atg7 siRNA was extracted using Trizol (Invitrogen). 2 μg RNA was used for reverse transcription RT, performed with Supercript III Kit (Invitrogen) according to the manufacturer's instructions. The quantitative PCR reaction for GFP was carried out with forward (5'-atggtgagcaagggcgaggagct) and reverse (5'-gaagatggtgcgctcctggacgt) primers and SYBR PCR mix (Applied Biosystems). In addition, parallel assays using the same cDNA pools were carried out using a Taqman MGB probe (Applied Biosystems) for glyceraldehyde-3-phosphate dehydrogenase (GAPDH). Data were captured on a 7900 HT Fast Real Time PCR System (Applied Biosystems) and analysed as a function of GAPDH expression.

Supplemental References

Dheda, K., Huggett, J.F., Bustin, S.A., Johnson, M.A., Rook, G., and Zumla, A. (2004). Validation of housekeeping genes for normalizing RNA expression in real-time PCR. Biotechniques *37*, 112-114, 116, 118-119.

Fass, E., Shvets, E., Degani, I., Hirschberg, K., and Elazar, Z. (2006). Microtubules Support Production of Starvation-induced Autophagosomes but Not Their Targeting and Fusion with Lysosomes. J Biol Chem *281*, 36303-36316.

Jahreiss, L., Menzies, F.M., and Rubinsztein, D.C. (2008). The Itinerary of Autophagosomes: From Peripheral Formation to Kiss-and-Run Fusion with Lysosomes. Traffic *9*, 574-587.

Kabeya, Y., Mizushima, N., Ueno, T., Yamamoto, A., Kirisako, T., Noda, T., Kominami, E., Ohsumi, Y., and Yoshimori, T. (2000). LC3, a mammalian homologue of yeast Apg8p, is localized in autophagosome membranes after processing. EMBO J *19*, 5720-5728.

Lawrence, B., and Brown, W. (1993). Inhibition of protein synthesis separates autophagic sequestration from the delivery of lysosomal enzymes. J Cell Sci *105*, 473-480.

Ravikumar, B., Imarisio, S., Sarkar, S., O'Kane, C.J., and Rubinsztein, D.C. (2008). Rab5 modulates aggregation and toxicity of mutant huntingtin through macroautophagy in cell and fly models of Huntington disease. J Cell Sci *121*, 1649-1660.

Sarkar, S., Davies, J.E., Huang, Z., Tunnacliffe, A., and Rubinsztein, D.C. (2007). Trehalose, a novel mTOR-independent autophagy enhancer, accelerates the clearance of mutant huntingtin and [alpha]-synuclein. J Biol Chem *282*, 5641-5652.

Figure S1. Proteasome substrates Ub^{G76V}-GFP and p53 have little or no dependence on autophagy for their clearance but inhibition of autophagy leads to impairment of Ub^{G76V}-GFP degradation.

(A) Knockdown of proteins involved in autophagy (Atg7 and Atg12) results in a decrease in autophagy. We assessed autophagosome numbers using the microtubule-associated protein 1 light chain 3 (LC3). LC3 is processed post-translationally into LC3-I, then converted to LC3-II, the only known protein that specifically associates with autophagosome membranes. LC3-positive vesicle numbers or LC3-II levels (versus actin) correlate with autophagosome numbers (Kabeya et al., 2000). siRNA knockdown in Hela cells stably expressing Ub^{G76V}-GFP was carried out as in Figure 1A and B.
(B) Increases in Ub^{G76V}-GFP levels in autophagy incompetent cells are not due to increases in transcription. mRNA levels were measured by quantitative PCR. Knockdown of Atg7 actually resulted in a decrease in mRNA levels of Ub^{G76V}-GFP relative to the housekeeping gene β-2-microglobulin (Dheda et al., 2004) (B2M-F tgctcgcgctactctctct, B2M-R tccattctctgctggatgac B2M-probe ctggaggctatccagcgtactccaa fam tamra labelled). Thus, the effects we see on protein levels may actually represent a greater decrease in protein turnover than the level of accumulation might suggest. Data shown are mean of four RNA extractions for each treatment, each quantified in triplicate.
(C) Treatment of cells with a range of chemical inhibitors of autophagy increases the levels of Ub^{G76V}-GFP. Cells were treated with 3-MA, or pepstatin A and E64d, for 48 h, or with bafilomycin A1 for 24 h, and the levels of Ub^{G76V}-GFP assessed by FACS analysis of GFP intensity.
(D) Ub^{G76V}-GFP, β-catenin and p53 are proteasome substrates. Inhibition of proteasome activity by MG132 in Ub^{G76V}-GFP Hela for 3 h leads to an accumulation of Ub^{G76V}-GFP, β-catenin and p53.
(E and F) Autophagy inhibition shows no effect on Ub^{G76V}-GFP, β-catenin and p53 levels. Treatment of Hela cells stably expressing Ub^{G76V}-GFP with the autophagy inhibitor bafilomycin A1 for 3h results in increased levels of the autophagy substrate p62 but no increase in Ub^{G76V}-GFP. Endogenous p53 and β-catenin levels are also unchanged. (F) shows densitometric quantification of three experiments, n.s. represents not significant, *** represents $p < 0.005$.
(G and H) Upregulation of autophagy has no effect on Ub^{G76V}-GFP or p53 levels and a small effect on β-catenin levels. Ub^{G76V}-GFP Hela were treated with 100 mM trehalose for 24 h to induce of autophagy. LC3-II levels in these cells increased (lower panel) demonstrating an increase in autophagosome number. No effect was seen on protein levels of Ub^{G76V}-GFP or p53, although levels of β-catenin were slightly reduced. We used the mTOR-independent autophagy inducer, trehalose, for these experiments as the mTOR inhibitor rapamycin inhibits translation and may cause spurious results. The experiment was carried out twice in triplicate with the same results, (H) shows densitometric analysis of one set of triplicate results. n.s. represents not significant, * represents $p < 0.05$.
(I and J) Treatment of cells with autophagy and proteasome inhibitors does not increase the levels of Ub^{G76V}-GFP more than those seen with proteasome inhibition alone as would be expected if autophagy were a route for clearance of this protein. Cells were treated with bafilomycin A1 for 32 h or Atg7 siRNA for 72 h at which time accumulation of Ub^{G76V}-GFP can be observed. Cells were treated with either lactacystin for the last 8 h (I), or MG132 for the last 3 h (J), and levels of Ub^{G76V}-GFP were assessed. Proteasome inhibitors were used for a short time

period to avoid toxicity of long-term treatment and to generate comparable levels of substrate accumulation with autophagy and proteasome inhibition. This would allow us to see any additional effect of autophagy inhibition over-and-above the proteasome inhibition. In the presence of proteasome inhibitor, treatment with bafilomycin A1 or Atg7 siRNA, has no further effect on accumulation of Ub^{G76V}-GFP suggesting it is not degraded by autophagy.

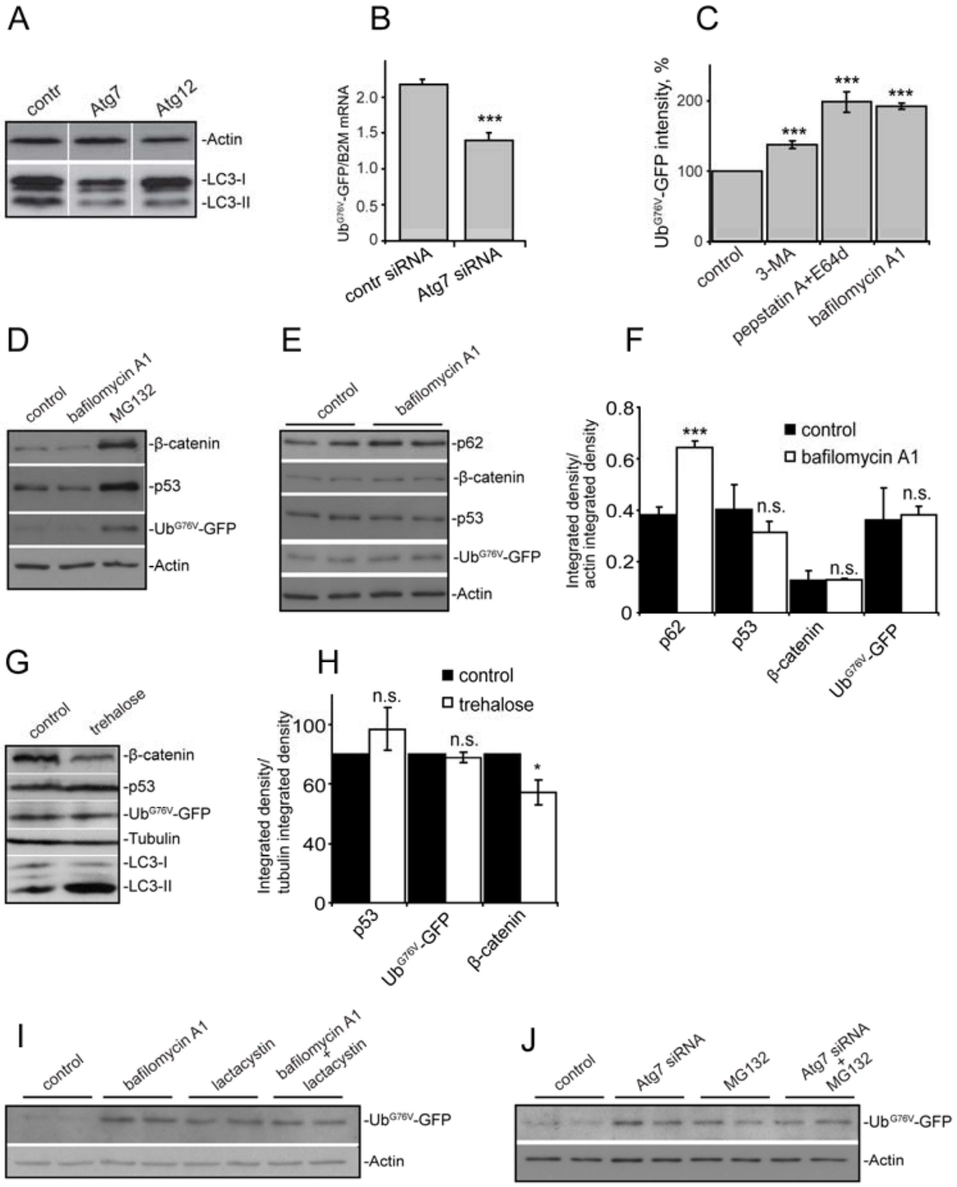

Figure S2. Knockdown of Atg7 increases levels of endogenous UPS substrates.

(A and B) Levels of β-catenin are increased in autophagy deficient cells. HeLa cells were transfected with 50 nM control or Atg7 siRNA followed by 72 h incubation to allow for protein knockdown. Cells were immunoblotted (A) and levels of β-catenin relative to actin were quantified (B).
(C and D) Treatment of cells with autophagy inhibitors (bafilomycin A1 or Atg7 siRNA) and proteasome inhibitors (MG132) does not increase the levels of p53 more than those seen with proteasome inhibition alone, as would be expected if autophagy were a route for clearance of this protein. The experimental paradigm was as described in Figure S1I and S1J above.
(E) p53 is degraded with delayed kinetics in autophagy impaired cells. Knockdown of Atg7 in HeLa cells was performed as in (A). Cells were incubated with 50 μg/ml cycloheximide, lysed at indicated time points and subjected to immunoblotting. Levels of p53 relative to actin were quantified by densitometry. Data on semi-log plot was fitted using one-phase non-linear regression.
(F) Half-life of p53 is increased in autophagy-impaired cells. Rate of decay of p53 as shown in (E) was analysed.
(G) Degradation of endogenous p53 is blocked in cells treated with proteasomal inhibitor MG132. HeLa cells were incubated with 50 μg/ml cycloheximide for the indicated periods in the presence or absence of proteasomal inhibitor MG132, then lysed and subjected to immunoblotting.

We assayed the turnover of a very short half-life protein in this cycloheximide chase experiment (as in main text Figure 2C), as protein synthesis inhibition for 3 h or more results in inhibition of autophagy (Lawrence and Brown, 1993). The short chase times (e.g. 15 min) in this experiment will almost certainly not affect putative p53 clearance via autophagy to any measurable extent, as the half-life of an autophagosome is about 20 min (Fass et al., 2006; Jahreiss et al., 2008). Any effect of cycloheximide on autophagy in this very short time-frame will also not lead to false conclusions. This may have a small effect in the autophagy-competent cells but will not have any effect in the autophagy-knockdown cells, so at worst, will only influence our results by lessening the effect size slightly.

For all the graphs, data are shown as means ± SE for three separate experiments. * $P < 0.05$ two-tailed t-test.

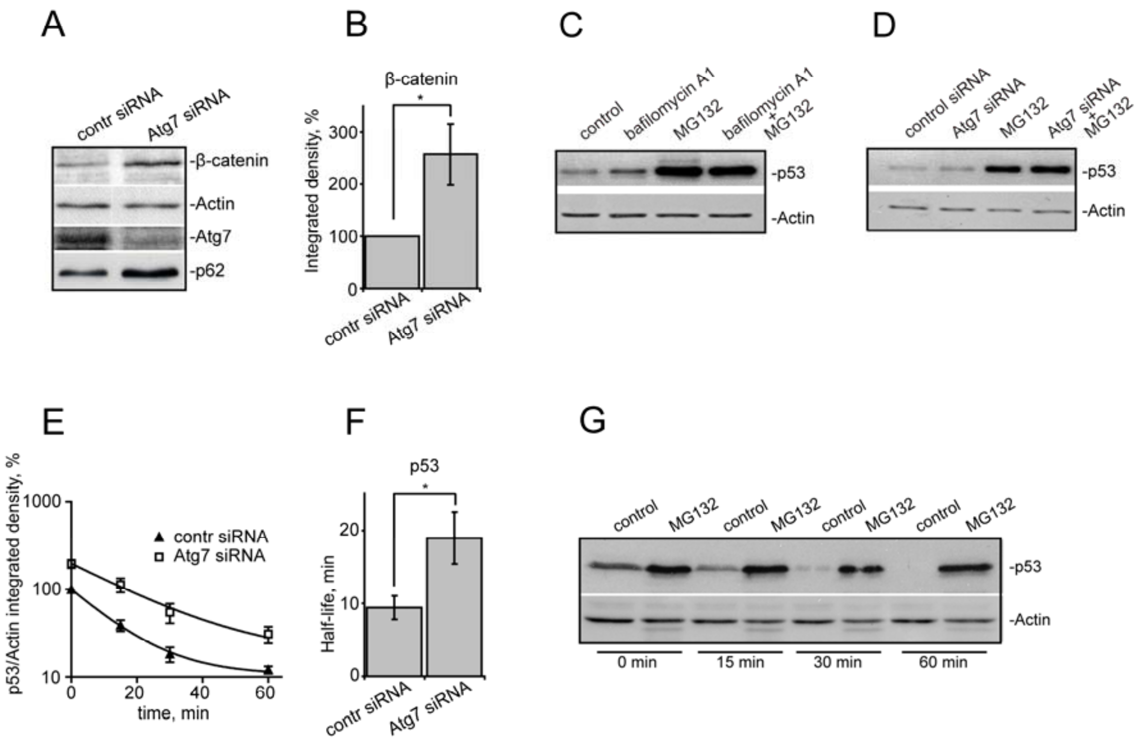
A
contr siRNA
Atg7 siRNA
-β-catenin
-Actin
-Atg7
-p62
B
β-catenin
Integrated density, %
300
200
100
0
contr siRNA
Atg7 siRNA
C
control
bafilomycin A1
MG132
bafilomycin A1 + MG132
-p53
-Actin
D
control siRNA
Atg7 siRNA
MG132
Atg7 siRNA + MG132
-p53
-Actin
E
p53/Actin integrated density, %
1000
100
10
0
20
40
60
time, min
contr siRNA
Atg7 siRNA
F
p53
Half-life, min
20
10
0
contr siRNA
Atg7 siRNA
G
control
MG132
-p53
-Actin
0 min
15 min
30 min
60 min

Figure S3. p62 does not sequester proteasome substrates in aggregates.

(A) Proteasome substrates accumulate in the soluble rather than insoluble cellular fraction following inhibition of autophagy. In order to establish if p62 prevented degradation of proteasome substrates by sequestering them in aggregates seen to form after inhibition of autophagy or the proteasome, we measured protein levels in the soluble and insoluble fractions. Ub^{G76V}-GFP HeLa were treated with bafilomycin A1 for 32 h or lactacystin for 8 h. Following this, cells were harvested and lysed in PBS supplemented with 0.5% Triton X-100 and 0.5% sodium deoxycholate. Following centrifugation (20 min, 13000 x g) the supernatant containing the soluble, cytoplasmic fraction was collected. The pellet (insoluble fraction) was resuspended in 1 x SDS-PAGE sample buffer and sonicated briefly. Protein concentration of the soluble fraction was measured by Bradford assay and diluted to equal concentrations; the insoluble fractions were diluted in the same ratio.

In bafilomycin A1 and lactacystin treated cells, p62 levels increase in the soluble fraction, but there is a dramatic increase in the levels of insoluble p62, particularly in bafilomycin A1 treated cells. This is not the case for Ub^{G76V}-GFP or p53, where no such increase is seen in the insoluble fraction with bafilomycin A1 treatment, although there is a slight increase of insoluble Ub^{G76V}-GFP in lactacystin treated cells.

(B) Ub^{G76V}-GFP and p53 do not form aggregates following autophagy inhibition. The presence of proteasome substrates in p62 aggregates was investigated by immunostaining and confocal microscopy. Hela cells stably expressing Ub^{G76V}-GFP were treated with bafilomycin A1 for 32 h to allow accumulation of p62. For the final 8 h of this treatment, lactacystin was added to allow Ub^{G76V}-GFP to accumulate to levels that can easily be observed by immunocytochemistry. Ub^{G76V}-GFP staining is mostly diffuse throughout the cytoplasm and there is no colocalisation seen with p62 aggregates (top panels).

For analysis of p53 localisation, cells were treated with bafilomycin A1 for 32 h. p53 staining can be seen throughout the cytoplasm and again does not co-localise with p62 aggregates (bottom panels). Scale bar applies to all images.

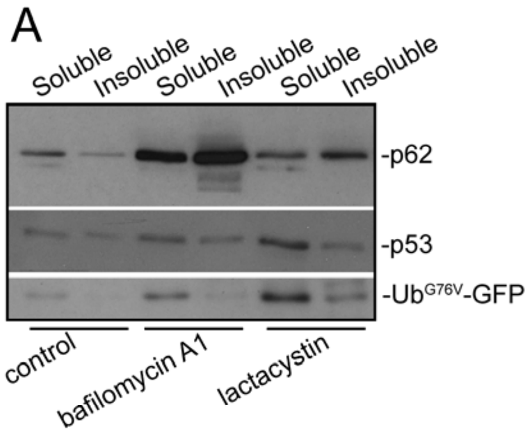
A
Soluble
Insoluble
Soluble
Insoluble
Soluble
Insoluble
-p62
-p53
-Ub^G76V-GFP
control
bafilomycin A1
lactacystin

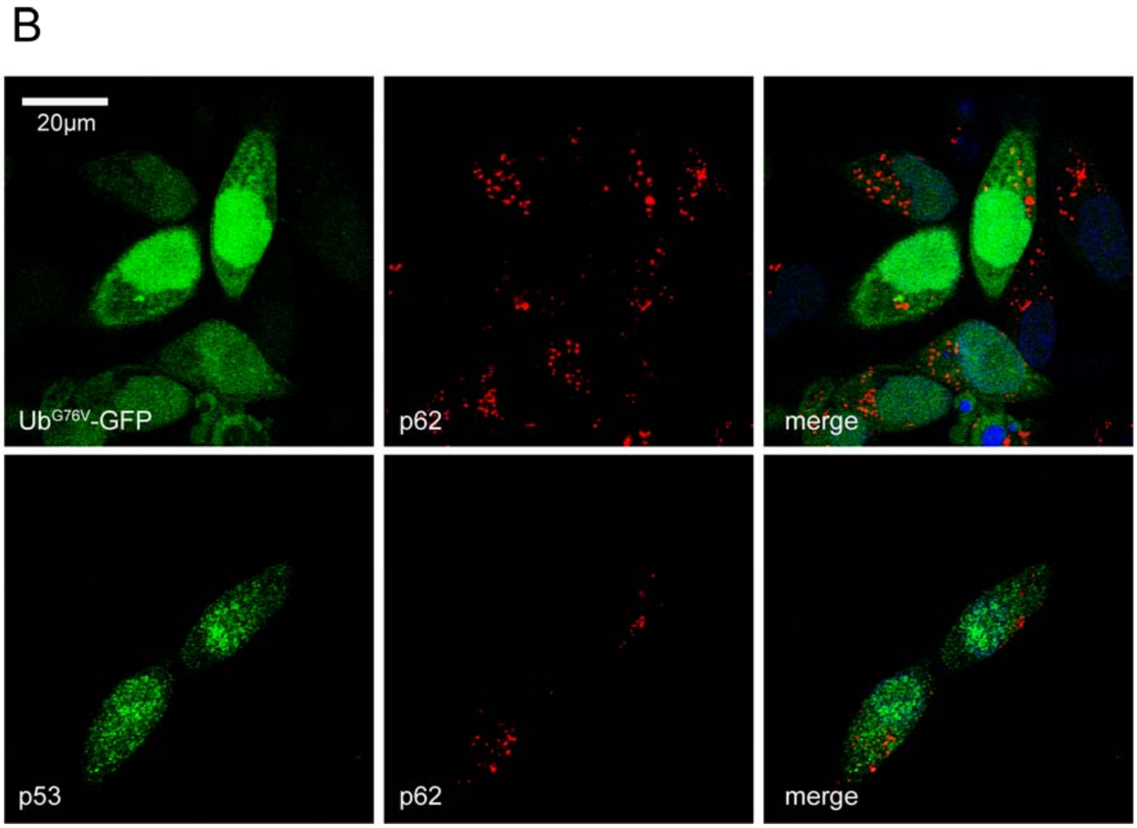
B
20µm
Ub^G76V-GFP
p62
merge
p53
p62
merge

Figure S4. Overexpression or knockdown of p62 does not affect autophagic activity.

(A) Overexpression of p62 does not affect LC3-II levels. HeLa cells were transfected with tdTomato-p62 or DsRed and grown for 48 h. Fluorescent cells were sorted, lysed and subjected to immunoblotting. LC3-II levels, used as a measurement of autophagy, were unchanged. Note, that after cell sorting, the amounts of LC3-I compared to LC3-II detected by the antibody, are low.
(B) LC3-II levels are not affected by knockdown of p62. HeLa cells were transfected with 100 nM control or p62-specific siRNA and incubated for 72 h. In the top panel cells were harvested without further treatment. In the lower panel, cells were incubated with bafilomycin A1 for 4 h before harvest to analyse autophagic flux. LC3-II accumulation can occur due to increased autophagosome formation or impaired autophagosome-lysosome fusion. Thus, we assayed LC3-II in the presence of bafilomycin A1, which blocks autophagosome-lysosome fusion, to assess autophagosome formation. Bafilomycin A1 increases LC3-II and the concentration used is saturating for LC3-II levels in this assay (Sarkar et al., 2007). We have previously demonstrated that autophagy-inducers can further increase LC3-II levels in bafilomycin A1-treated cells, indicating that such compounds increase autophagosome formation (Sarkar et al., 2007). Conversely, we have shown that inhibition of autophagosome synthesis reduces LC3-II levels in bafilomycin A1 treated cells.

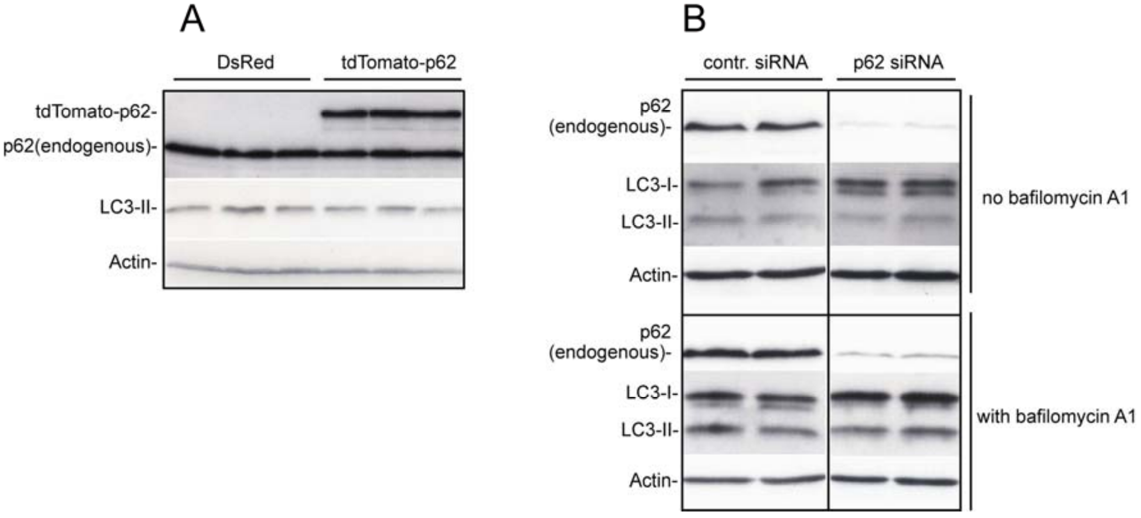

Figure S5. p62 increases polyQ aggregates only when proteasomal degradation is not inhibited.

(A) htt Q74 overexpression does not inhibit UPS flux. In order to assess the effects of htt Q74 overexpression on the ubiquitin proteasome system, Ub^{G76V}-GFP levels were measured following overexpression of hemagglutinin epitope-tagged htt Q74. Expression of this protein for 48 h has been shown to result in formation of intracellular aggregates (Ravikumar et al., 2008) but had no effect on Ub^{G76V}-GFP levels as analysed by FACS.

(B) p62 overexpression increases polyQ aggregation and toxicity. HEK293 cells were co-transfected with EGFP-httQ74 and either DsRed (control) or tdTomato-p62 at 1:3 ratios. The percentage of green/red double positive cells with EGFP-httQ74 aggregates or cell death was assessed 48 h post-transfection.

(C) p62 increases polyQ aggregation and toxicity in cells where autophagy but not the proteasome is inhibited. HEK293 cells were transfected as in (B) followed by 24 h incubation in 10 mM 3-MA or 10 μM lactacystin. Cells were fixed and polyQ aggregation and cell death quantified.

(D) p62 does not increase polyQ aggregation in cells where both autophagy and proteasome are inhibited. SK-N-SH cells where cotransfected with EGFP-httQ74 and either DsRed (control) or tdTomato-p62 at 1:3 molar ratios followed by 24 h (no further treatment) or 12 h incubation in 10 mM 3-methyladenine (3-MA) and 10 μM lactacystin. Cells were fixed and polyQ aggregation assessed.

(E) p62 overexpression increased aggregation of ubiquitin-primed polyQ but not when proteasome activity is inhibited. SK-N-SH cells where cotransfected with Ubi-R-Q112-EGFP and either DsRed (control) or tdTomato-p62 at 1:3 molar ratios followed by 48 h (no further treatment) or 12 h (10 μM lactacystin) incubation. The percentage of green/red double positive cells with Ubi-R-Q112-EGFP aggregates was assessed.

For all the graphs, data are shown as means ± SE for three separate experiments. *** $P < 0.005$ two-tailed t-test.

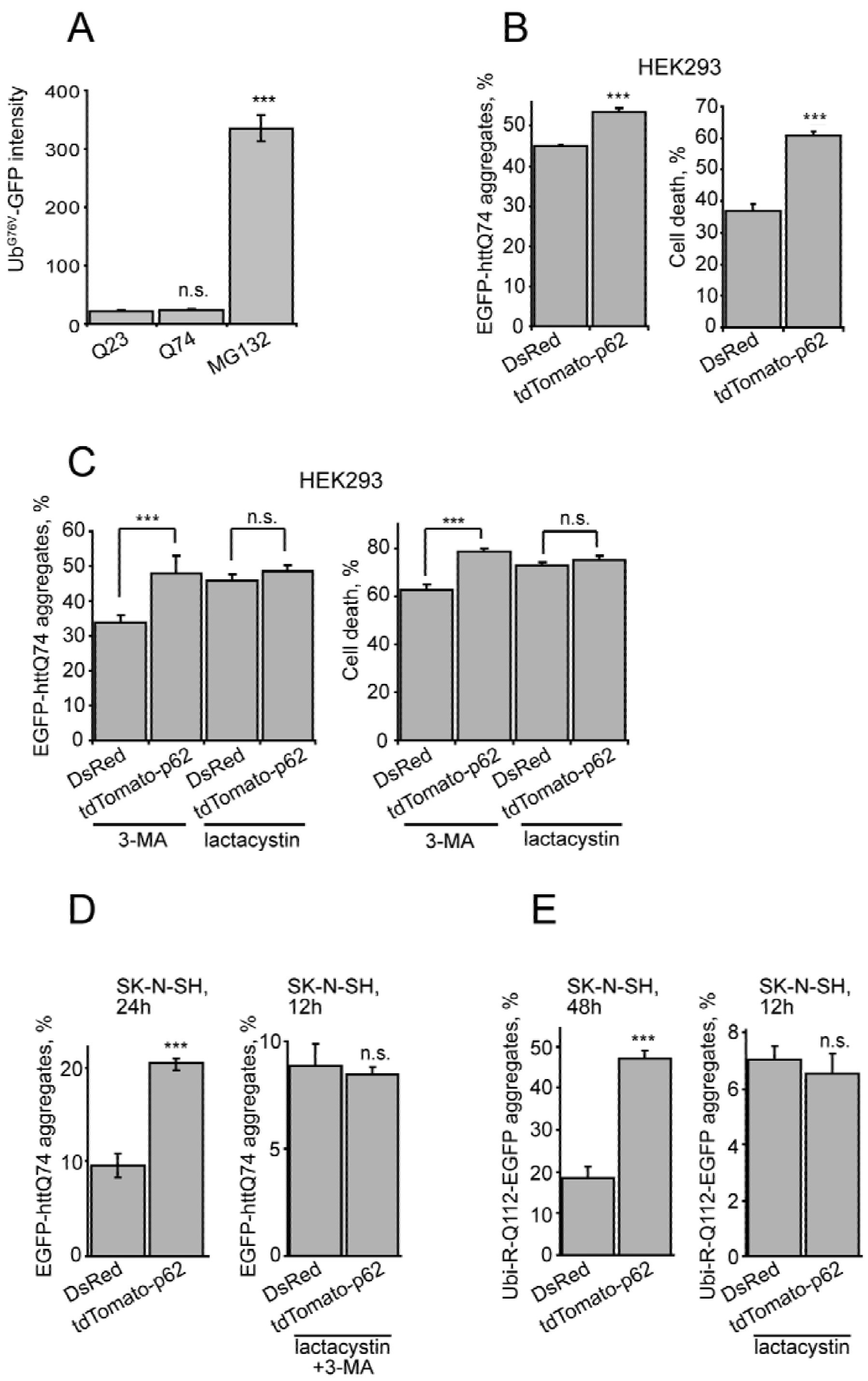
A
Ub^{G76V}-GFP intensity
400
300
200
100
0
***
n.s.
Q23
Q74
MG132
B
HEK293
EGFP-httQ74 aggregates, %
50
40
30
20
10
0
***
DsRed
tdTomato-p62
Cell death, %
70
60
50
40
30
20
10
0
***
DsRed
tdTomato-p62
C
HEK293
EGFP-httQ74 aggregates, %
60
50
40
30
20
10
0
***
n.s.
DsRed
tdTomato-p62
DsRed
tdTomato-p62
3-MA
lactacystin
Cell death, %
80
60
40
20
0
***
n.s.
DsRed
tdTomato-p62
DsRed
tdTomato-p62
3-MA
lactacystin
D
SK-N-SH, 24h
EGFP-httQ74 aggregates, %
20
10
0
***
DsRed
tdTomato-p62
SK-N-SH, 12h
EGFP-httQ74 aggregates, %
10
5
0
n.s.
DsRed
tdTomato-p62
lactacystin +3-MA
E
SK-N-SH, 48h
Ubi-R-Q112-EGFP aggregates, %
50
40
30
20
10
0
***
DsRed
tdTomato-p62
SK-N-SH, 12h
Ubi-R-Q112-EGFP aggregates, %
8
6
4
2
0
n.s.
DsRed
tdTomato-p62
lactacystin

Figure S6. Knockdown of p62 does not rescue direct inhibition of proteasomal activity.

(A) HeLa cells stably expressing Ub^{G76V}-GFP were transfected with 50 nM control or p62 siRNA. After 72 h cells were left untreated or incubated with 10 µM MG132 for 16 h. The GFP fluorescence intensity was quantified by FACS.
(B) Cells as in (A) were lysed and analysed by immunoblotting.
For all the graphs, data are shown as means ± SE for two separate experiments performed in triplicate. n.s. denotes not significant.

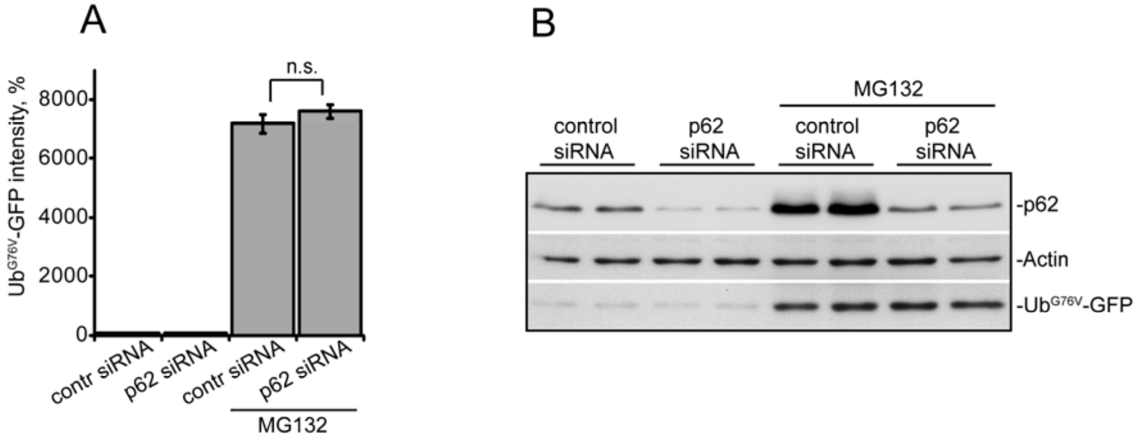

Figure S7. Ubiquitin positive p62 aggregates accumulate following inhibition of either autophagy or proteasome.

HeLa cells were treated with bafilomycin A1 or MG132 for 16 h. Immunostaining was carried out using anti-p62 antibody (BD transduction labs 1:500), with anti-mouse secondary antibody conjugated to Alexa 488 (Invitrogen) and anti-ubiquitin antibody (Dako 1:1000) with anti-rabbit secondary antibody conjugated to Alexa 594 (Invitrogen). Cells were imaged by confocal microscopy. Both p62 and ubiquitin positive aggregates can be seen to accumulate to high levels in cells treated with either inhibitor. Scale bar applies to all panels.

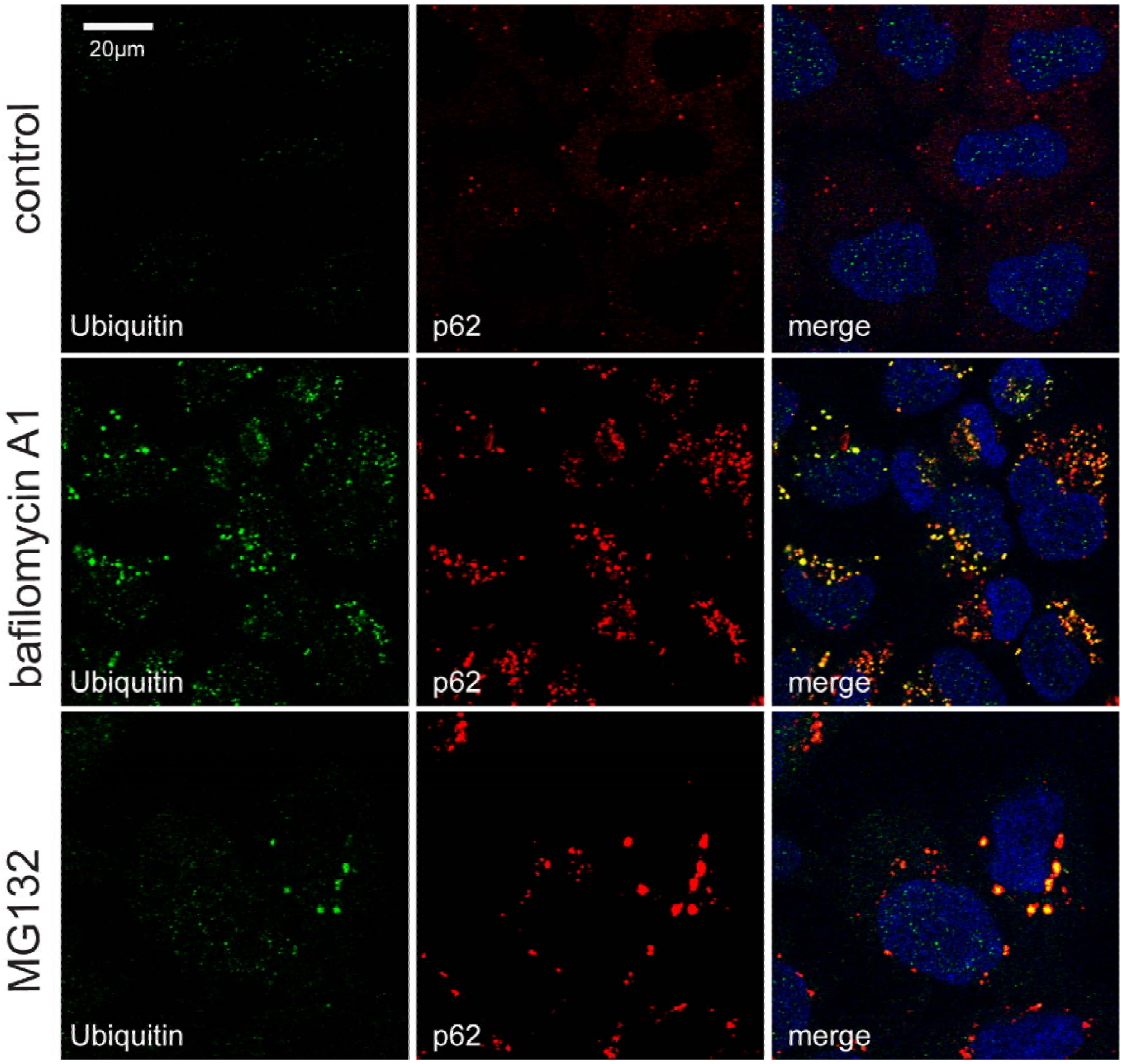

Figure S8. htt Q74 aggregates are distinct from ubiquitin and p62 aggregates that form when autophagy is impaired.

(A and B) HeLa cells were transfected with httQ74-GFP with or without bafilomycin A1 treatment. After 24h cells were fixed and immunostained for p62 (A) or ubiquitin (B) as for Figure S7. Whilst both p62 and ubiquitin antibodies labelled the Q74 aggregates a distinct pool of p62- or ubiquitin-positive aggregates was seen to accumulate following bafilomycin treatment that did not contain httQ74-GFP.

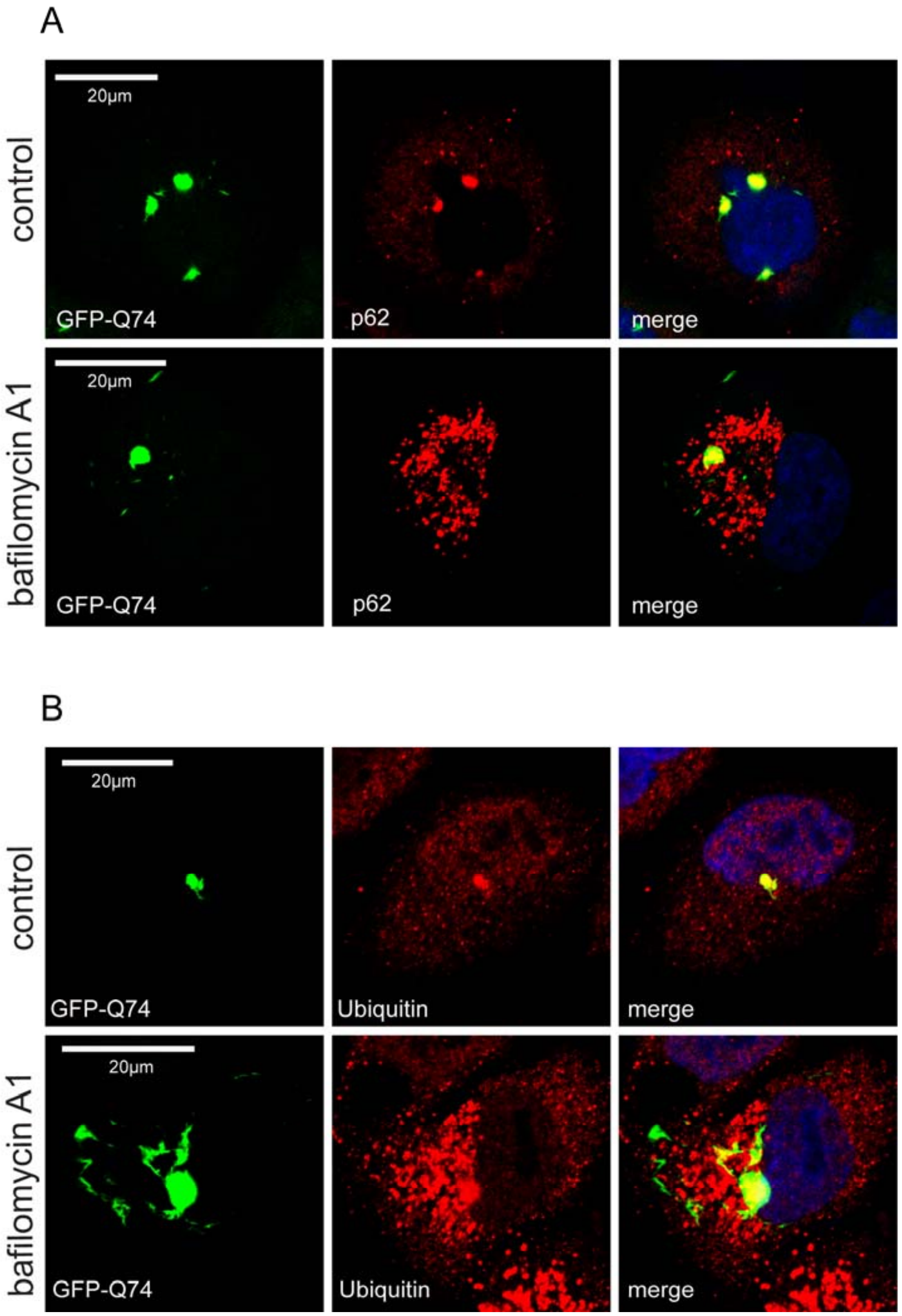

Supplement: Document S1.Supplemental Experimental Procedures and Eight Figures [file mmc1.pdf]
